# Antibacterial and residual antimicrobial activities against *Enterococcus faecalis* biofilm: A comparison between EDTA, chlorhexidine, cetrimide, MTAD and QMix

**DOI:** 10.1038/srep12944

**Published:** 2015-08-06

**Authors:** Rui Zhang, Min Chen, Yan Lu, Xiangjun Guo, Feng Qiao, Ligeng Wu

**Affiliations:** 1 Department of Endodontics and Restorative Dentistry, School of Stomatology, Tianjin Medical University, Tianjin, China; 2 Department of Maxillofacial Surgery, School of Stomatology, Tianjin Medical University, Tianjin, China

## Abstract

We compared the antibacterial and residual antimicrobial activities of five root canal irrigants (17% EDTA,2% chlorhexidine,0.2% cetrimide, MTAD, and QMix) in a model of *Enterococcus faecalis* biofilm formation. Sixty dentin blocks with 3-week *E. faecalis* biofilm were divided into six equal groups and flushed with irrigant for 2 min or left untreated. A blank control group was also established. Antibacterial activities of the irrigants were evaluated by counting colony forming units. To test residual antimicrobial activities, 280 dentin blocks were divided into seven equal groups and flushed with irrigant for 2 min or left untreated and then incubated with *E. faecalis* suspension for 48 h, or used as a blank. No bacteria were observed in the blank control group. The number of viable *E. faecalis* was significantly fewer in the irrigant-treated groups compared with the untreated control (*P* < 0.05). Among the five irrigants, QMix had the strongest antibacterial activity. Residual antimicrobial activities of CHX were significantly higher at 12 h, 24 h and 36 h compared to untreated control (*P* < 0.05). All five root canal irrigants were effective to some extent against *E. faecalis*, but QMix and CHX had the strongest, and CHX the longest (up to 36 h), antimicrobial activity.

The root canal biofilm is the main reason for root canal treatment failure^1^. Many studies have shown that *Enterococcus faecalis* is the predominant bacterial species in teeth with persistent periapical periodontitis^2^,^3^,^4^. *Enterococcus faecalis* is detected in 24%–77% of the filled root canals with persistent periapical lesions and usually exists in the infected root canals in the form of a biofilm^5^. This species is also present in most peripheral parts of the root-canal system, such as fins, anastomoses, apical canals, lateral canals, and dentinal canals^6^,^7^. The smear layer, which is formed only in instrumented areas, enhances the adhesion of *E. faecalis* and provides a matrix for deeper bacterial infection, thus influencing the effect of root canal irrigants on bacterial removal^8^. Therefore, *E. faecalis* biofilm has strong viability and drug resistance.

Several root canal irrigants have antimicrobial activities when contacting microorganisms directly, but few can eliminate *E. faecalis* absolutely^9^. Researchers have proved that 17% EDTA, 2% chlorhexidine (CHX), and 0.2% cetrimide (CTR) can eliminate *E. faecalis* at some extent^10^,^11^. However, the effects of CHX on *E. faecalis* are weaker than those of MTAD and Tetraclean^12^. MTAD (a mixture of tetracycline isomer, citric acid, and detergent) can sterilize *E. faecalis* rapidly, as can 0.2% CHX^13^,^14^. CTR is also a cationic surfactant with excellent antibacterial activity^15^. However, the residual antimicrobial activity of CHX could last longer than that of CTR^16^. QMix, a recent root canal irrigant that was first introduced in the marked in 2012^17^, has comparable scavenging effects on smear layer to EDTA and its antibacterial effect is better than that of CHX^18^.

Currently, most studies focus on the antibacterial activities of different irrigating solutions. However, there are few studies on the residual antimicrobial activities of root canal irrigants. Therefore, this study aimed to compare the antibacterial and residual antimicrobial activities of five root canal irrigants (17% EDTA, 2% CHX, 0.2% CTR, MTAD, and QMix) in a model of *E. faecalis* biofilm infection of dentin blocks.

## Material and Methods

### Preparation of the dentin blocks

A total of 200 freshly extracted, intact maxillary and mandibular molars without preoperative treatment, root caries, and cracks were collected from the maxillofacial department of Tianjin Medical University Dental Hospital. The study was approved by the Medical Ethics Committee of Tianjin Medical University and the methods were carried out in accordance with the Declaration of Helsinki (2008). All patients have obtained the informed consent.

The dental calculus and soft tissue remnants of all molars were mechanically cleaned with a periodontal curette, and then stored in a 0.1% thymol solution at 4 °C until required. Next, a total of 350 dentin blocks (2 mm × 2 mm × 1 mm [width × length × height]) were prepared by selecting the coronal one-third of the roots (from the cementoenamel junction); then, the pulpal side of the dentin blocks was polished with 600-grit silicon carbide abrasive paper (Buehler, IL, USA). The dentin blocks were immersed into 3% NaOCl (Septodont, Saint-Maur, France) for 1 min, and then transferred into 17% EDTA (META, Chungbuk, Republic of Korea) for 2 min to remove the smear layer. Next, the dentin blocks were steam autoclaved for 30 min under 15 psi pressure at 121 °C to ensure that no bacteria remained. All samples were preserved in 37 °C brain heart infusion (BHI; Qingdao Haibo Biotechnology Co., Ltd., Qingdao, China) for 24 h to test for the presence of bacteria. After confirming complete sterilization, all dentin blocks were put into sterile saline.

### Preparation of the bacterial suspension

After the standard strains of *E. faecalis* preserved at −80 °C were thawed, they were inoculated into freshly prepared BHI and cultivated under anaerobic condition at 37 °C for 24 h. Then, a bacterial suspension with 1 × 10^7^/ml *E. faecalis* was obtained by dilution with BHI broth.

### Root canal irrigants

Five root canal irrigants were used in the present study: 17% EDTA (Sigma-Aldrich, St Louis, USA), 2% CHX (Sigma-Aldrich), 0.2% CTR (Sigma-Aldrich), MTAD (Dentsply Tulsa, Tulsa, OK), and QMix (Dentsply Tulsa, Tulsa, OK).

### Antibacterial activity test

A total of 60 dentin blocks were randomly selected from the initial 350 blocks and put into 200 μl tubes (one block per tube). Then, 180 μl *E. faecalis* suspension was added to each tube. After sealing with Parafilm®, the tubes were incubated under aerobic condition at 37 °C for 3 weeks. During this period, BHI broth was changed every other day to maintain normal growth of *E. faecalis*. Before each exchange, two samples of broth culture were collected randomly. A portion from each sample was inoculated into BHI, and further cultivated at 37 °C for 24 h. Finally, some of the bacterial colonies were dyed with Gram stain. Finally, the *E. faecalis*-infected models were established.

The 60 infected dentin blocks were flushed with 180 μl sterile saline for 2 min, and put into 96-well plates (one block per well). The dentin blocks were randomly divided into six groups: EDTA, CHX, CTR, MTAD, QMix, and untreated control groups (n = 10 per group); ten sterile dentin blocks were used as the blank control group. The five different root canal irrigants or BHI broth were added into the corresponding wells (100 μl per well). After 2-min incubation, the dentin blocks were removed and dried with aseptic absorbent paper. Next, the dentin blocks were centrifuged in 200 μl BHI for 2 s and vortexed (Vortex-6, Shanghai Pure One Biotechnology Co., Ltd., Shangai, China) for 1 min. The supernatants in the centrifuge tubes were removed by pipette and diluted ten times. The diluted supernatants were inoculated into BHI medium and cultivated under anaerobic conditions at 37 °C for 24 h. Finally, the colony-forming units (CFUs) were counted.

### Residual antimicrobial activity test

The remaining 280 sterile dentin blocks were randomly divided into seven groups: EDTA, CHX, CTR, MTAD, QMix, untreated control, and blank control groups (n = 40 per group). The dentin blocks were put into 96-well plates (one block per well). The five different root canal irrigants or BHI broth were added into the corresponding wells (100 μl per well). All blocks were removed after 2 min and put into 200-μl tubes. *Enterococcus faecalis* suspension (180 μl) was added to the treated and untreated control groups, while an equal volume of BHI broth was added to the blank control group.

All samples were incubated at 37 °C and cultivated under anaerobic conditions for 48 h. Every 12 h, ten samples were collected from each group to count the CFUs using the same method as described above.

### Statistical analysis

After the values of CFUs were converted into lgCFUs, data expressed as mean ± standard deviation were analyzed by SPSS version 17.0 statistical software (Chicago, IL, USA). The differences in the antibacterial and residual antimicrobial activities were analyzed by one-way and multivariate ANOVA. A *P*-value of less than 0.05 was considered to be statistically significant.

## Results

### Antibacterial activity

**Figure 1:**
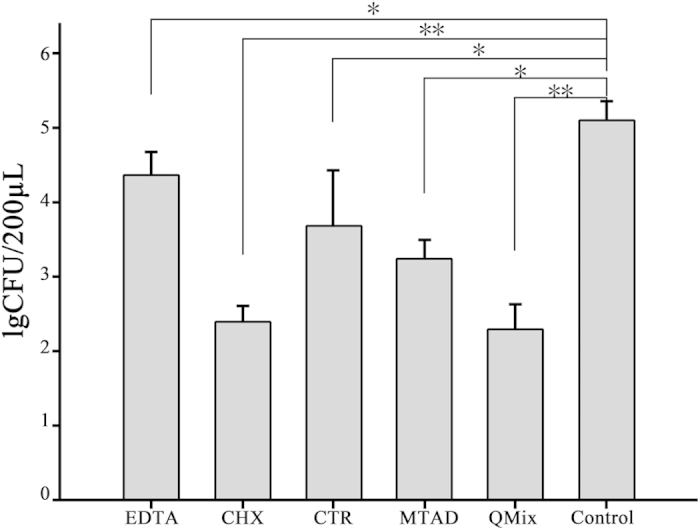
Antibacterial activities (lgCFUs) of the five root canal irrigants (EDTA, CHX, CTR, MTAD, and QMix) and untreated control groups. *P < 0.05, **P < 0.01 compared with the untreated control group.

No bacteria were observed in the blank control group. Compared with the untreated control group, *E. faecalis* IgCFUs in dentin blocks treated with EDTA, CHX, CTR, MTAD, and QMix were significantly reduced (*P* < 0.05) (Table 1, Fig. 1). The QMix group had the lowest lgCFU value (2.31 ± 0.32), followed by the CHX group (2.41 ± 0.21), the MTAD group (3.26 ± 0.23), the CTR group (3.71 ± 0.72), and the EDTA group (4.37 ± 0.31). The antibacterial activities of the six groups were significantly different (*P* < 0.05), except between CHX and QMix groups (*P* > 0.05).

### Residual antimicrobial activity

**Figure 2:**
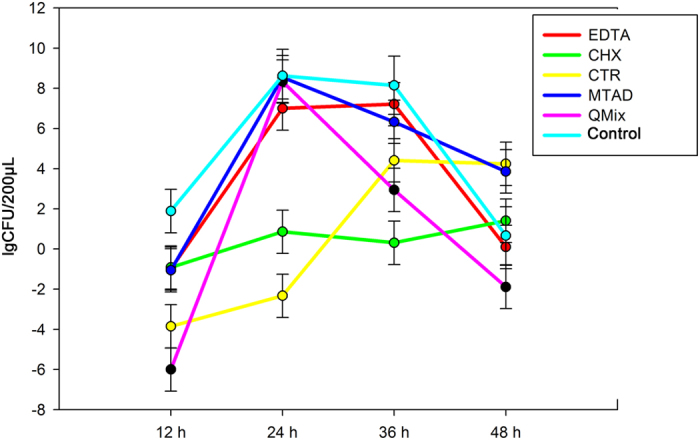
Residual antimicrobial activities (lgCFUs) of the five root canal irrigants (EDTA, CHX, CTR, MTAD, and QMix) and untreated control groups at 12 h, 24 h, 36 h, and 48 h.

Differences in the residual antimicrobial activities among the six groups at 12 h, 24 h, 36 h, and 48 h are shown in Table 1 and Fig. 2. Compared with the untreated control group, the residual antimicrobial activities of the EDTA, CHX, and MTAD groups were not significantly different (*P* > 0.05). On the other hand, the lgCFUs of the CTR and QMix groups were significantly lower than those of the untreated control group (−3.85 ± 2.89 [CTR] and −6.00 ± 0.00 [QMix] vs. 1.89 ± 0.14 [untreated control]; *P* < 0.05) at 12 h. There was no significant difference in the residual antimicrobial activities among the EDTA, MTAD, QMix, and the untreated control groups at 24 h (*P* > 0.05). However, the lgCFUs of the CHX and CTR groups were significantly lower than those of the untreated control group (0.86 ± 6.51 and −2.33 ± 5.50 vs. 8.62 ± 0.02; *P* < 0.05) at 24 h. At 36 h, only the lgCFUs of the CHX group was significantly lower than those of the untreated control group (0.31 ± 4.72 vs. 8.15 ± 0.09; *P* < 0.05). There was no significant difference in the residual antimicrobial activities among the treated and untreated control groups at 48 h (*P* > 0.05).

## Discussion

*Enterococcus faecalis*, which usually exists in the form of a biofilm, is considered one of the most intractable bacteria. Kishen *et al.* have determined the chemical composition of *E. faecalis* biofilms and observed apatite deposition on mature biofilm^19^. This microstructure enhanced *E. faecalis* adhesion and drug resistance, making it more difficult to remove. This study was designed to identify the most effective root canal irrigant against *E. faecalis*, in the hopes of improving root canal treatment outcomes.

We discovered that 2% CHX had similar antibacterial activity to QMix. It has been reported that 2% CHX can sterilize 99.93% of the bacteria in *E. faecalis* biofilm^10^. QMix is composed of various ingredients, including EDTA, CHX, and detergents. As the amounts of QMix and 2% CHX were the same in this study, QMix contained relatively less CHX. However, CHX in QMix might produce synergistic antibacterial activity with EDTA, and the detergents in QMix might increase wettability and permeability, which might altogether enhance the antibacterial activity of QMix. Therefore, the antibacterial activities of 2% CHX and QMix were similar.

EDTA has not shown an obvious antibacterial effect against *E. faecalis* in many studies^10^,^20^. This study indicated that the antibacterial activity of 17% EDTA against *E. faecalis* was weaker than that of the other four irrigants. As a chelating agent removing the inorganic components in the smear layer, EDTA could change the permeability of cell membrane; this is the mechanism for its antibacterial activity^21^. Nevertheless, 17% EDTA has large surface tension and small permeability, which made it difficult to penetrate into the dentine tubules to kill the *E. faecalis* exhaustively.

Previous studies have shown that 0.1% CTR and 2% CHX could sterilize similar amount of *E. faecalis* on the dentin^13^,^22^. In this study, the antibacterial effect of 0.2% CTR was weaker than that of 2% CHX, which might be due to the fact that CTR had a stronger ability of reducing bacterial adhesion, so more viable bacteria could be counted.

Residual antimicrobial activities of the five irrigants were also examined to evaluate their effects on inhibiting *E. faecalis* biofilm formation. At 12 h, the fewest viable bacteria were detected in the QMix group, followed by the CTR group. However, at 24 h, the number of viable bacteria in QMix group increased significantly, suggesting that residual antimicrobial activity of QMix was strong, yet of short duration. Although there were more viable bacteria at 24 h than at 12 h, the CTR group had the lowest CFUs at 24 h, and residual antimicrobial activity of CTR remained until at least 36 h. Therefore, residual antimicrobial activity of CTR was more stable and durable.

Residual antimicrobial activities of irrigants weaken gradually and finally disappear^23^, as confirmed in this study except for CHX. CHX did not have residual antimicrobial activity at 12 h, while its residual antimicrobial activity became significant at 24 h and 36 h, probably because there were few *E. faecalis* at 12 h or CHX had a weak residual antimicrobial activity. Since the number *E. faecalis* increased gradually, but the residual antimicrobial activity of CHX did not change over time, the effect of CHX became gradually decreased. Therefore, compared with CTR and QMix, CHX had the weakest yet the most stable residual antimicrobial activity. Baca *et al.* have tested the residual antimicrobial activities of CHX and CTR with a fresh bacteria suspension and found that CHX and CTR can last for more than 40 days^16^. In this study, the residual antimicrobial activities of the five irrigants disappeared at 48 h. In our study, bacteria were grown in a closed system (also known as a batch culture) to which no nutrients were added or removed, thus maintaining a constant culture volume. The growth curve of a typical batch culture can be divided into four phases: lag phase, log phase, stationary phase, and decline phase. Eventually, the death rate of *E. faecalis* gradually increases as nutrients are depleted and toxic waste products accumulate, and *E. faecalis* ultimately enter the decline phase. Residual antimicrobial activities of EDTA and MTAD may not have been detected because of this experimental limitation. In addition, a relatively large volume of bacterial suspension was added, and the small amount of irrigating solution remaining on the dentin blocks was diluted, which would decrease its residual antimicrobial activity.

In conclusion, EDTA, CHX, CTR, MTAD, and QMix can kill *E. faecalis* in the matured biofilm at different levels. Their antibacterial activity from strong to weak was CHX and QMix, MTAD, CTR, and EDTA. However, only CHX, CTR, and QMix had residual antimicrobial activities that lasted for at least 36 h, 24 h, and 12 h, respectively.

## Additional Information

**How to cite this article**: Zhang, R. *et al.* Antibacterial and residual antimicrobial activities against *Enterococcus faecalis* biofilm: A comparison between EDTA, chlorhexidine, cetrimide, MTAD and QMix. *Sci. Rep.* **5**, 12944; doi: 10.1038/srep12944 (2015).

## Figures and Tables

**Table 1:** Antibacterial and residual antimicrobial activities (lgCFUs) of the five root canal irrigants (EDTA, CHX, CTR, MTAD, and QMix) and untreated control groups.

Groups	Antibacterial activity	Residual antimicrobial activity			
		12 h	24 h	36 h	48 h
EDTA	4.37 ± 0.31^*^	−0.98 ± 3.78	7.00 ± 0.18	7.21 ± 0.06	0.10 ± 4.58
CHX	2.41 ± 0.21^*^	−0.92 ± 2.92	0.86 ± 6.51^*^	0.31 ± 4.72^*^	1.41 ± 2.80
CTR	3.71 ± 0.72^*^	−3.85 ± 2.89^*^	−2.33 ± 5.50^*^	4.41 ± 0.23	4.24 ± 0.25
MTAD	3.26 ± 0.23^*^	−1.06 ± 3.72	8.55 ± 0.03	6.33 ± 0.07	3.86 ± 0.42
QMix	2.31 ± 0.32^*^	−6.00 ± 0.00^*^	8.34 ± 0.03	2.94 ± 5.08	−1.89 ± 4.88
Untreated	5.09 ± 0.26	1.89 ± 0.14	8.62 ± 0.02	8.15 ± 0.09	0.66 ± 6.08

CFUs: colony-forming units; EDTA: ethylene diamine tetraacetic acid; CHX: chlorhexidine; CTR: cetrimide; MTAD: a mixture of tetracycline isomer, citric acid and detergent.

^*^P < 0.05 compared with the untreated control group.
